# Mobile Accelerometer Applications in Core Muscle Rehabilitation and Pre-Operative Assessment

**DOI:** 10.3390/s24227330

**Published:** 2024-11-16

**Authors:** Aleš Procházka, Daniel Martynek, Marie Vitujová, Daniela Janáková, Hana Charvátová, Oldřich Vyšata

**Affiliations:** 1 Department of Mathematics, Informatics and Cybernetics, University of Chemistry and Technology in Prague, 166 28 Prague 6, Czech Republic; daniel.martynek@vscht.cz; 2 Czech Institute of Informatics, Robotics and Cybernetics, Czech Technical University in Prague, 160 00 Prague 6, Czech Republic; 3 Department of Sports Medicine, 2nd Faculty of Medicine and FN Motol, Charles University in Prague, 150 00 Prague 5, Czech Republic; marie.vitujova@fnmotol.cz (M.V.); daniela.janakova@fnmotol.cz (D.J.); 4 Centre for Security, Information and Advanced Technologies (CEBIA-Tech), Faculty of Applied Informatics, Tomas Bata University in Zlín, 760 01 Zlín, Czech Republic; charvatova@utb.cz; 5 Department of Neurology, Faculty of Medicine in Hradec Králové, Charles University in Prague, 500 05 Hradec Králové, Czech Republic; oldrich.vysata@fnhk.cz

**Keywords:** physical activity monitoring, motion symmetry, rehabilitation, abdominal wall repair, computational intelligence, accelerometers, machine learning

## Abstract

Individual physiotherapy is crucial in treating patients with various pain and health issues, and significantly impacts abdominal surgical outcomes and further medical problems. Recent technological and artificial intelligent advancements have equipped healthcare professionals with innovative tools, such as sensor systems and telemedicine equipment, offering groundbreaking opportunities to monitor and analyze patients’ physical activity. This paper investigates the potential applications of mobile accelerometers in evaluating the symmetry of specific rehabilitation exercises using a dataset of 1280 tests on 16 individuals in the age range between 8 and 75 years. A comprehensive computational methodology is introduced, incorporating traditional digital signal processing, feature extraction in both time and transform domains, and advanced classification techniques. The study employs a range of machine learning methods, including support vector machines, Bayesian analysis, and neural networks, to evaluate the balance of various physical activities. The proposed approach achieved a high classification accuracy of 90.6% in distinguishing between left- and right-side motion patterns by employing features from both the time and frequency domains using a two-layer neural network. These findings demonstrate promising applications of precise monitoring of rehabilitation exercises to increase the probability of successful surgical recovery, highlighting the potential to significantly enhance patient care and treatment outcomes.

## 1. Introduction

Human activity recognition [1,2] and artificial intelligence (AI) [3] have a wide range of applications in rehabilitation, neurology, and sports. Wearable sensors are widely used in individual physiotherapy, detection of various types of pain, and improvements in physical fitness [4]. Specialized rehabilitation exercises and physical activities [5] play a crucial role in the pre-operative and post-operative stages of surgical treatment [6,7] to optimize the healing and recovery process. This area is increasingly important in line with population ageing, with demands for general surgery expected to rise. However, post-operative complications following abdominal surgery are frequently reported.

Pre-operative assessment is crucial for optimizing surgical outcomes and minimizing post-operative complications, despite the overall success rate sometimes being limited [8]. Traditional pre-operative evaluation methods primarily rely on medical history, physical examination, and imaging studies, which may not always capture the dynamic physiological changes occurring in a patient’s daily activities. Various studies show positive effects of rehabilitation in patients undergoing orthopedic surgery [9] and on recovery after abdominal surgery [10]. Deep learning models have been developed to predict rare but severe post-operative complications following specific surgical treatments [11,12].

Different studies focus on prehabilitation exercises and their evaluation [13,14,15,16] by computational methods, and their monitoring via telemedicine equipment [17,18] to reduce complication rates and risk factors associated with complex abdominal surgeries. Recent advancements in inertial measurement units (IMUs) for motion capture have introduced novel approaches to pre-operative assessment [7,19,20] and general rehabilitation, with thermal cameras [21] and mobile accelerometers emerging as promising tools in this domain. Mobile accelerometers, commonly found in smartphones and wearable devices [22,23,24,25], can continuously monitor a patient’s movements, providing real-time data on physical activity, posture, and mobility. This wealth of information offers a unique opportunity to enhance the pre-operative assessment process, enabling a more comprehensive understanding of a patient’s functional capacity and aiding surgeons in tailoring their decisions and approach to abdominal wall repair.

The use of mobile accelerometers in pre-operative assessment allows for the collection of objective and quantitative data on a patient’s movement patterns and activity levels. By continuously monitoring these metrics, healthcare providers can gain insights into a patient’s functional capacity, identify specific movement patterns, and pinpoint high-risk patients. Various rehabilitation programs [26,27,28] study the effectiveness of specific exercises involving repetitive muscle contractions, core stability, and balance exercises. Further studies explore the use of force sensors to monitor respiratory functions and measure the activation of abdominal wall muscles [29].

Utilizing mobile accelerometers for core muscle rehabilitation and pre-operative assessment involves two key phases: preparing the patients for surgery by assessing their core strength and stability, and aiding in their post-surgery recovery by monitoring and guiding their rehabilitation. By integrating wearable sensors with artificial intelligence tools, clinicians can now assess and monitor patient movements more precisely, allowing for personalized rehabilitation plans.

Mobile accelerometers can play a significant role in both pre-operative and post-operative rehabilitation. By tracking a patient’s progress in real-time, rehabilitation protocols can be personalized, monitored remotely, and adjusted based on the patient’s response to therapy. This enables more efficient and effective rehabilitation, potentially reducing recovery time, minimizing complications, and improving overall patient satisfaction.

Data processing methods are based on the general methodology of signal processing [30], computational intelligence [31], and time-frequency signal analysis. This approach evaluates features for assessing the balance criterion associated with individual rehabilitation exercises. The classification of symmetry in rehabilitation [32,33] can vary depending on the specific motion parameters [34] being considered and the clinical context. Evaluating separate rehabilitation exercises based on the development of sensor technology has been crucial in realizing the potential for both clinical and remote rehabilitation. While there is no fixed number of symmetry degrees universally used, an objective scoring system can be proposed to evaluate the feasibility of balance assessment technology for adaptation into remote rehabilitation settings. Specific exercises can be proposed for both prehabilitation before different kinds of abdominal surgeries and to treat various motion disorders [35,36,37], utilizing the important research area of body kinematics [38].

The integration of mobile accelerometers into the rehabilitation and pre-operative assessment process [39] has the potential to enhance interaction between patients, rehabilitation specialists, and surgeons. By leveraging this technology, healthcare providers can gain deeper insights into a patient’s daily life and functional capabilities, enabling them to make more informed decisions and provide personalized care. As we explore the role of mobile accelerometers in the context of complex abdominal surgeries [40,41,42,43], including open, robotic, and laparoscopic techniques, we will uncover the transformative impact they can have on patient outcomes and the future of surgical practice.

There are many factors that influence ody motion symmetry and asymmetrical movement patterns. They mainly include injuries, muscle strength, age, and neurological disorders that can impact muscle control and coordination, resulting in asymmetry due to impaired movement on one side of the body.

The goal of this paper is to discuss the benefits of using mobile accelerometers in the evaluation of rehabilitation exercises to reduce the probability of complications after surgeries. Associated topics include the abilities of this methodology to capture objective and quantitative data, track changes in physical activity levels, and detect movement patterns. Another goal is to address the challenges and limitations associated with this technology, such as data privacy concerns, device compatibility, and the need for standardized algorithms to interpret accelerometer data. The proposed data analysis procedures contribute to this research area by (i) demonstrating the use of smartphones, communication links, and remote data stores to record accelerometric data during rehabilitation exercises, (ii) proposing a fast symmetry level evaluation using a suggested global criterion function, and (iii) designing a general web-page that allows data import, remote signal processing in both time and frequency domains, and evaluation of the coefficient of symmetry. The novelty of the paper lies in the use of communication links for data acquisition with remote storage and the proposal of a symmetry coefficient evaluation.

## 2. Methods

**Figure 1:**
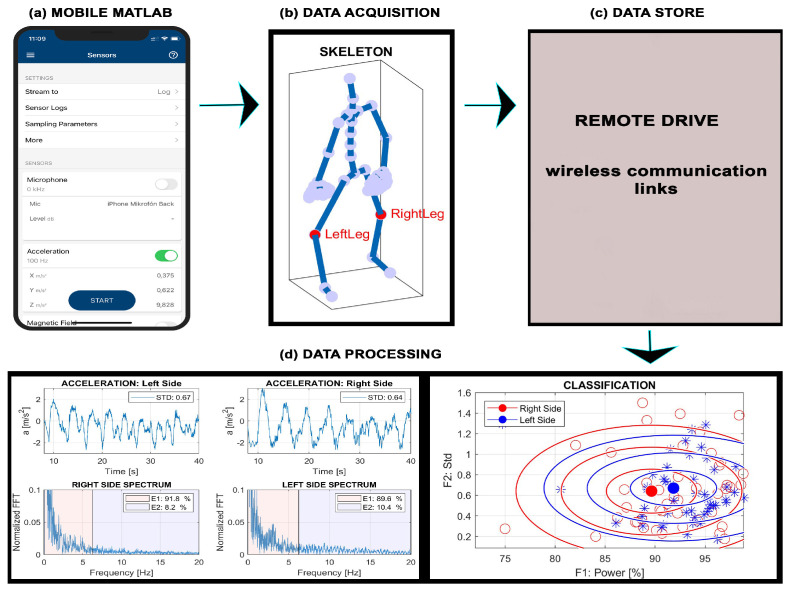
Principle of data processing during rehabilitation exercises presenting (**a**) mobile Matlab initialization, (**b**) data acquisition using accelerometric sensors inside the smartphone, (**c**) export of recorded signals to the remote drive, and (**d**) processing of data on the remote drive in time and frequency domains to extract motion features and evaluate the coefficient of symmetry.

This paper describes the use of wearable accelerometers to analyze the symmetry of different rehabilitation exercises using wearable sensors embedded in a mobile phone [44,45] placed on the body. Figure 1 illustrates the framework for analyzing rehabilitation exercises and data processing that include

(a) Activation of sensors in a smartphone and specification of their parameters in the mobile Matlab environment.
(b) Data acquisition from the right and left part of the body with the selected sampling frequency.
(c) Export of signals through communication links into the remote drive.
(d) Evaluation of accelerometric signals, estimation of the symmetry coefficient of left/right parts of the body, and classification of motion features.

The selected rehabilitation exercises were acquired and processed in the Matlab 2024b (MathWorks, Natick, MA, USA) computational environment. Data were recorded by mobile Matlab connected to the Matlab cloud with saving data on the Matlab Drive.

The dataset includes records acquired by a smartphone equipped with a three-axis accelerometer. All procedures involving human participants were conducted in accordance with the ethical standards of the institutional research committee and the 1964 Helsinki Declaration and its later amendments. The study received ethical approval from the Ethics Committee (UCT EK/7/2022), and the anonymity of the obtained data was strictly maintained.

The analysis is based on eight exercises performed during 1280 tests on different individuals. Detailed descriptions of observations can be found on IEEE DataPort (Rehabilitation Exercises and Computational Intelligence, 10.21227/xp41-7325) [46] for further investigation. This repository includes the accelerometric data acquired during all experiments, an informative video presentation of the rehabilitation exercises, the Matlab graphical user interface, and a graphical video abstract of the paper.

### 2.1. Data Acquisition

**Figure 2:**
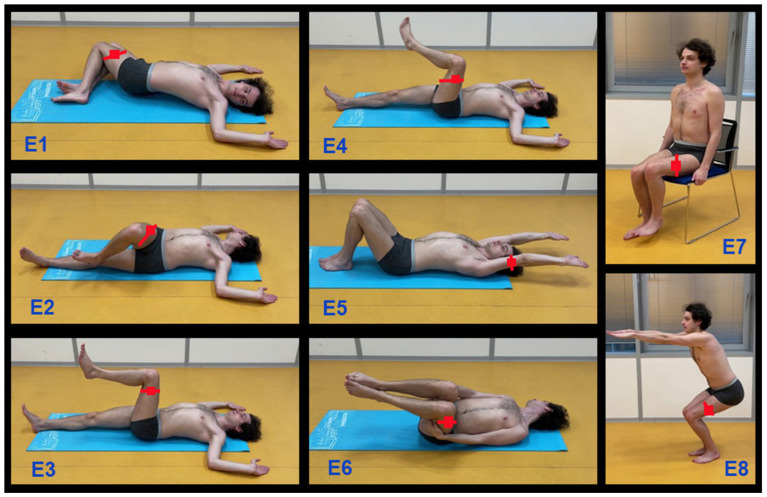
Selected rehabilitation exercises used for accelerometric data acquisition recorded by wearable sensors (red squares) located on the left and right sides of the body used for data acquisition and processing in the computational and visualization environment of the mobile Matlab system.

Figure 2 and Table 1 present a brief specification of the selected rehabilitation exercises. The smartphone was affixed to the left or right leg or arm with the display facing forward [47] and was used as a sensor for accelerometric data acquisition via the mobile Matlab application, with a sampling frequency of 100 Hz. Signals from the left and right sides of the body were acquired and processed in the Matlab environment. Each exercise was repeated ten times and performed during 16 tests involving different individuals.

The study included 16 participants, comprising 9 males and 7 females, with ages ranging from 8 to 75 years and BMI value 23.4±2.9 kg/m^2^. Detailed participant information is presented in Table 2.

### 2.2. Signal Processing

In the field of rehabilitation, accelerometric data processing is essential for monitoring and analyzing movement patterns. Computational intelligence tools play a significant role in this domain by providing advanced methods for data analysis, interpretation, and decision-making, which aid in developing personalized rehabilitation programs.

Fundamental signal processing methods include digital filtering techniques to remove noise and extract relevant signal components. The Fourier transform converts accelerometric signals from the time domain to the frequency domain, facilitating the analysis of periodic components. Alternatively, the Wavelet transform offers multi-resolution analysis of accelerometric signals, enabling the detection of both transient and continuous features. Machine learning algorithms, such as support vector machines, decision trees, and neural networks, are then employed to classify movement patterns and predict rehabilitation outcomes.

Three-dimensional accelerometers are widely used in rehabilitation to monitor and assess body movement comprehensively, using acceleration data across three orthogonal axes (*x*, *y*, and *z*). The resulting data, observed with the sampling frequency $f_{s}$, form column vectors $d_{p}$ for position *p*, representing the left (p=L) and right (p=R) parts of the body. Each sequence can then be divided into *M* subsequences, each *N* values long, defining a vector $\left[d_{L}^{\left(1\right)},\cdots ,d_{L}^{\left(M\right)},d_{R}^{\left(1\right)},\cdots ,d_{R}^{\left(M\right)}\right]$ and forming a time-domain signal matrix:

(1) $$D_{N\times Q}\;=\;\left[\begin{array}{cccc} d_{1} & d_{2} & \cdots & d_{Q} \end{array}\right]$$

with Q=2×M columns. The elements of each column vector $d_{q}={\left\{d_{q}\left(n\right)\right\}}_{n=(k-1)N+1}^{kN}$ for q=1,2,⋯,Q specify the observed accelerometric data.

By applying the discrete Fourier transform (dft) to each column of the matrix $D_{N\times Q}$, we can construct the associated matrix $G_{N\times Q}$ as follows:

(2) $$G_{N\times Q}=dft\left(D_{N\times Q}\right)$$

This matrix contains the frequency components of the separate signal subwindows in its column vectors, with frequency components ranging from 0 to $f_{s}$ Hz. The values in each column *q* of the matrix $G_{N\times Q}$ are evaluated by the following relation:

(3) $$g_{q}\left(k\right)=\sum_{n=1}^{N}\left(d_{q}\left(n\right)-{\bar{d}}_{q}\right)\;e^{-j(k-1)(n-1)2\pi /N}\;$$

for k=1,2,⋯,N, and the mean value ${\bar{d}}_{q}$ of each column *q* of the matrix $D_{N\times Q}$ for a selected individual and the left and right sides of the body. Spectral components are evaluated with the frequency resolution $\frac{1}{N}f_{s}$ Hz and its values $f\left(k\right)=\frac{k}{N}f_{s}$.

With the frequency components of each accelerometric signal subwindow, it is possible to evaluate the relative power $E_{q}^{\left(i\right)}$ for each subwindow *q* of the observed sequence in the frequency band $B^{\left(i\right)}=\langle f_{c_{1}}^{\left(i\right)},f_{c_{2}}^{\left(i\right)}\rangle$ using spectral components evaluated by the discrete Fourier transform according to Equation (3) by the relation:

(4) $$E_{q}^{\left(i\right)}=\frac{\sum_{n\in \Phi^{\left(i\right)}}{\left|g_{q}\left(n\right)\right|}^{2}}{\sum_{n=1}^{N/2}{\left|g_{q}\left(n\right)\right|}^{2}}$$

where $\Phi^{\left(i\right)}$ is the set of indices for the frequency components $f{\left(k\right)}^{\left(i\right)}\in \langle f_{c_{1}}^{\left(i\right)},f_{c_{2}}^{\left(i\right)}\rangle$. This process can be applied for the whole matrix $G_{N\times Q}$ to find *Q* features of separate subwindows and selected frequency bands.

When analyzing a selected rehabilitation exercise, each subwindow can be described (i) in the time domain by its mean and standard deviation, and (ii) in the frequency domain by the power in the selected frequency bands. This forms a matrix with time- and frequency-domain features for the left and right side of the body forming a vector $\left[p_{L}^{\left(1\right)},\cdots ,p_{L}^{\left(M\right)},p_{R}^{\left(1\right)},\cdots ,p_{R}^{\left(M\right)}\right]$.

Matrix $D_{N\times Q}$ is used for the evaluation of means and standard deviations in the time domain, as well as to evaluate power components to form a pattern matrix:

(5) $$\begin{array}{c} P_{R\times Q}\;=\left[p_{1},p_{2},\cdots ,p_{Q}\right] \end{array}\;$$

that includes *R* features evaluated for each subwindow associated with the rehabilitation experiment.

The associated target vector specifying the positions of sensors includes the values [L,⋯,L,R,⋯,R] that can be substituted by target probabilities of each class:

(6) $$T_{S\times Q}=\left[t_{1},t_{2},\cdots ,t_{Q}\right]=\left[\begin{array}{cccccc} 0 & \cdots & 0 & 1 & \cdots & 1\\ 1 & \cdots & 1 & 0 & \cdots & 0 \end{array}\right]$$

for classification into two classes (*S* = 2) associated with the positions of the sensors on the body during each rehabilitation exercise.

Motion symmetry is a valuable concept in analyzing rehabilitation exercises because it allows clinicians and researchers to evaluate whether movements on both sides of the body are aligned, balanced, and coordinated, which is essential for assessing the recovery progress. During rehabilitation, symmetry in motion between the left and right sides of the body is often a goal, particularly before and after surgeries using specific motion capture systems or wearable sensors.

Complete records of the accelerometric signal for the left and right sides of the body were divided into *M* segments and for each of them, the evaluation was performed to define the left-side feature $F_{q}^{\left(L\right)}\left(r\right)$ and the right-side feature $F_{q}^{\left(R\right)}\left(r\right)$ based on property *r* for segment q=1,2,⋯,M. The symmetry index, based on a commonly used one, can be calculated by the following relation:

(7) $$c_{q}\left(r\right)=\frac{1}{2}\;\frac{F_{q}^{\left(L\right)}\left(r\right)-F_{q}^{\left(R\right)}\left(r\right)}{F_{q}^{\left(L\right)}\left(r\right)+F_{q}^{\left(R\right)}\left(r\right)}\;100$$

The average of $c_{q}\left(r\right)$ over all segments q∈〈1,Q〉 results in the standard symmetry coefficient related to the selected feature *r*.

The alternative criterion for one experiment and a selected feature set can be evaluated using the proposed relation:

(8) $$C=\sqrt{\frac{1}{length(\Psi )}\;\sum_{r\in \Psi }\frac{1}{Q}\sum_{q=1}^{Q}c_{q}\left(r\right)}\;$$

where Ψ is the selected set of features using the global symmetry criterion evaluation.

Classifying rehabilitation exercises using accelerometers involves several steps, including data collection, preprocessing, feature extraction, and classification. The classification of *Q* signal segments by a specific machine learning method typically requires the determination of the pattern and target matrices. In this case, the pattern matrix $P_{R,Q}$ defined by Equation (5), and target matrix $T_{S,Q}$ specified by Equation (6), respectively, were used. The number of features was reduced to R=2 for better visualization.

Commonly used algorithms for signal segment classification include support vector machines (SVM), which are effective in high-dimensional spaces, Bayesian methods, and the simple and commonly used *k*-nearest neighbor methods. Alternatively, neural network methods, including deep learning approaches, are suitable for handling large and complex systems. In the simplest case of a two-layer neural network with $S_{1}$ and $S_{2}$ elements in the first and second layers, respectively, the outputs $A_{S_{1},Q}^{\left(1\right)}$ and $A_{S_{2},Q}^{\left(2\right)}$ of the individual layers are evaluated by the following relations:

(9) $$\begin{array}{ccc} A_{S_{1},Q}^{\left(1\right)}\;\; & = & \;\;f_{1}\left(W_{S_{1},R}^{\left(1\right)}\;P_{R,Q},b_{S_{1},1}^{\left(1\right)}\right)\\ A_{S_{2},Q}^{\left(2\right)}\;\; & = & \;\;f_{2}\left(W_{S_{2},S_{1}}^{\left(2\right)}\;A_{S_{1},Q}^{\left(1\right)},b_{S_{2},1}^{\left(2\right)}\right)\; \end{array}$$

with the network coefficients forming matrices $W_{S_{1},R}^{\left(1\right)}$ and $W_{S_{2},S_{1}}^{\left(2\right)}$, and vectors $b_{S_{1},1}^{\left(1\right)}$ and $b_{S_{2},1}^{\left(2\right)}$. The proposed model uses the sigmoidal transfer function $f_{1}$ in the first layer and the probabilistic softmax transfer function $f_{2}$ in the second layer.

To determine the predictive model’s ability to perform classification during practical implementation, the *k*-fold cross-validation method is often used. In this paper, the leave-one-out method, with the same number of folds as the number of data points, is employed.

When implementing and evaluating models, it is crucial to consider the context and the specific costs associated with false positives and false negatives. Sensitivity and specificity provide a clear picture of the model’s performance in identifying both positive and negative cases, aiding in making decisions about model deployment and potential improvements. For classifying rehabilitation exercises using accelerometers into two classes, common performance metrics can be used:

- Sensitivity (True positive rate, recall) defined as the proportion of actual positives that are correctly identified by relation:
  (10) $$TPR=\frac{TP}{TP+FN}\;$$
- Specificity (True negative rate) defined as the proportion of actual negatives that are correctly identified by relation:
  (11) $$TNR=\frac{TN}{TN+FP}\;$$
- Accuracy defined as a probability of global correct classification:
  (12) $$AC=\frac{TP+TN}{TP+TN+FP+FN}\;$$

where TP, TN, FP and FN stand for the number of true positive, true negative, false positive, and false negative classifications [48].

## 3. Results

**Figure 3:**
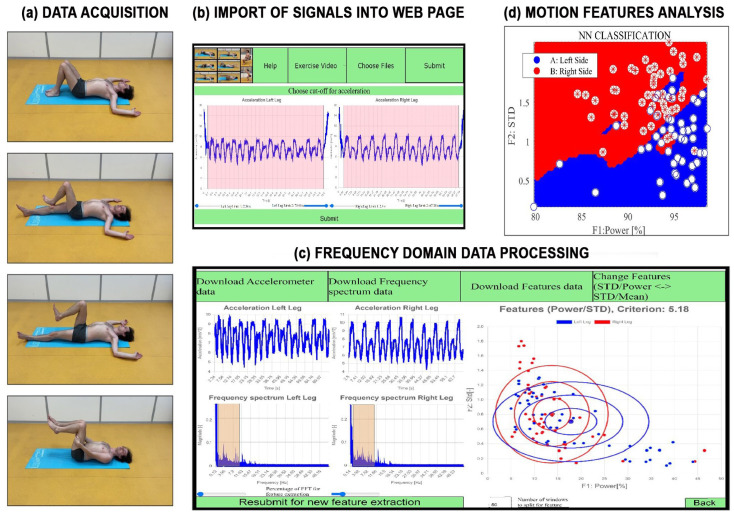
Principle of data processing during rehabilitation exercises presenting (**a**) animation of motion exercises to train individuals and data acquisition using a smartphone, (**b**) data import into the proposed web-page, (**c**) frequency domain remote signal processing including symmetry coefficient estimation, and (**d**) extraction and analysis of motion features.

The proposed graphical user interface [49,50] is presented in Figure 3. It enables the visualization of rehabilitation exercises through videos accessible from the initial web-page. The motion accelerometric data acquisition and processing using a specific web-page includes the following steps:

- Animating motion exercises for training and data acquisition by a mobile phone.
- Selecting accelerometric signals recorded by the smartphone of a chosen individual and stored in the specified datastore.
- Trimming inaccurate data at the beginning and end of each record.
- Evaluating spectral components recorded on the right and left sides of the body using the discrete Fourier transform, with results displayed in Figure 3b.
- Estimating the percentage power of signals in selected frequency ranges and specified subwindows.
- Visualizing motion features associated with the left and right sides of the body.
- Evaluating the proposed symmetry criterion coefficient for the selected rehabilitation exercise.

Table 3 lists the individuals, types of rehabilitation exercises, and the symmetry criterion values evaluated by Equation (8) using features in the frequency domain for each exercise. The last column includes the average symmetry index values for each individual. The last two rows present the overall average symmetry coefficients across all individuals and their standard deviations.

**Figure 4:**
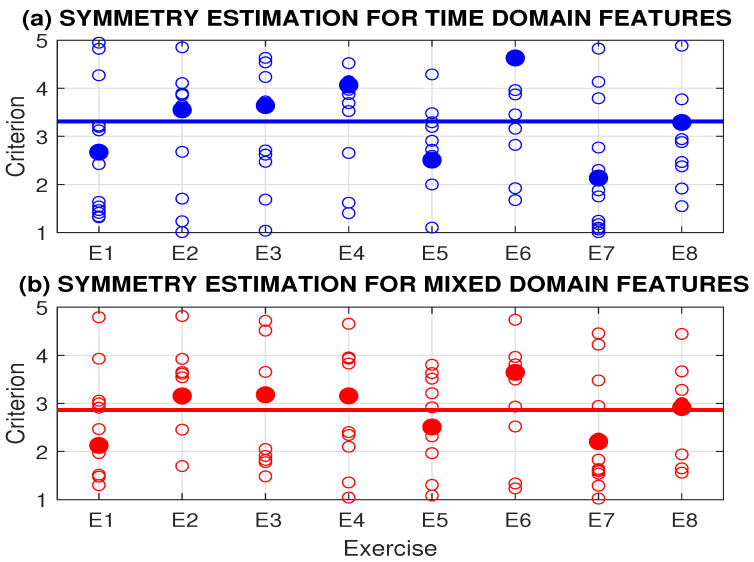
Symmetry criteria for 8 rehabilitation exercises evaluated by (**a**) time domain and (**b**) mixed-domain features presenting mean values by 16 tests of different individuals with 10 repetitions of each rehabilitation exercise.

Figure 4 presents the symmetry criteria for eight rehabilitation exercises, evaluated using both time domain and spectral domain features. It shows the mean values of 16 tests, each with 10 repetitions of each rehabilitation exercise for a selected individual. The highest asymmetry, exceeding the mean value, was observed for exercises E2, E3, E4, and E6 by both methods. The best symmetry criterion coefficients were observed for exercises E1 and E7.

**Figure 5:**
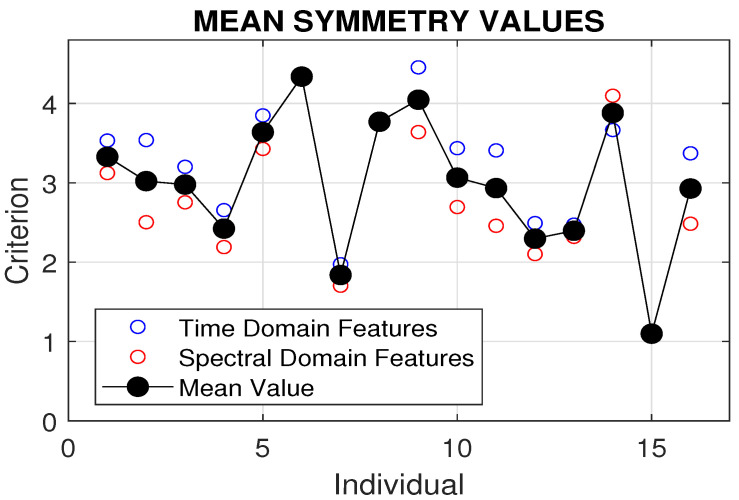
Comparison of symmetry criteria for 16 tests involving different individuals and eight rehabilitation exercises, evaluated using time domain and spectral domain features.

More detailed results are presented in Table 3 for the set of individuals under study. A comparison of symmetry criteria for 16 tests involving different individuals and 8 rehabilitation exercises, evaluated using time domain and spectral domain features, is shown in Figure 5. The best symmetry was observed for individual 15, with a mean symmetry coefficient of 1.1 across all rehabilitation exercises.

**Figure 6:**
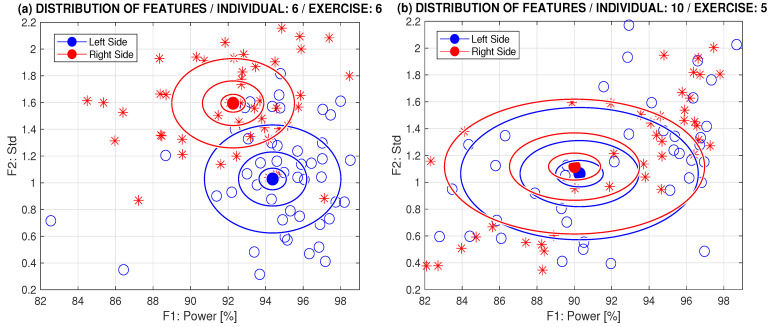
Comparison of distribution of the time and spectral domain features for selected exercises of (**a**) prevailing asymmetric motion (individual 6, exercise 6) and (**b**) prevailing symmetric motion (individual 10, exercise 5) with centers of the right and left side positions and *c* multiples of standard deviations for c=0.2,0.5,1.

Features evaluated from the sensors on the left and right sides of the body for selected exercises, as well as mixed-domain features, are presented in Figure 6. This comparison shows results for selected exercises that demonstrate prevailing asymmetric and symmetric motions, with centers of the right and left side positions and multiples of standard deviations for c=0.2,0.5,1.

**Figure 7:**
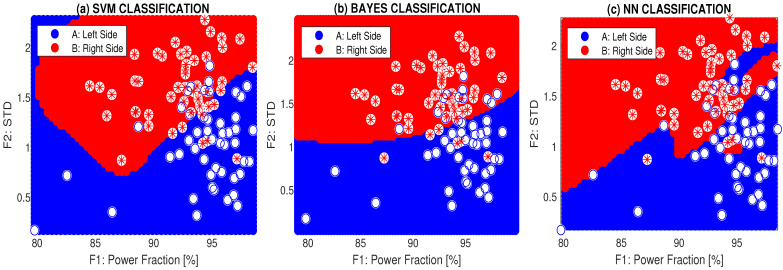
Classification of symmetry features of the body cross-motion by mixed features using (**a**) support vector machine, (**b**) the Bayes method, and (**c**) the two-layer neural network for a selected individual 6-DH.

Figure 7 presents the classification of the symmetry features for cross-motion (exercise 6) using mixed features and three different methods: support vector machine, the Bayesian method [51,52], and a two-layer neural network (NN) with 10 neurons in the first layer and sigmoidal/softmax transfer functions in the first and second layers, respectively.

A summary of the accuracy and cross-validation errors for all individuals, a selected rehabilitation exercise, and different classification methods is presented in Table 4. The highest classification accuracy of 90.6% for individual 6-DH corresponds with their worst coefficient of symmetry in Table 3. The cross-validation errors were calculated using the leave-one-out method.

## 4. Discussion

This paper focuses on evaluating rehabilitation exercises designed to strengthen the abdominal wall and reduce complications during potential surgeries. Accelerometric data from a variety of exercises were used for symmetry analysis of different motion patterns across 16 individuals. The features obtained were evaluated in both the time and spectral domains to classify rehabilitation segments and various exercises. This approach is significant for improving body fitness levels, reducing potential chest pains, and serving as pre-operative muscle training before open or robotic surgery [53].

The most important features for evaluating the rehabilitation exercises were based on signals recorded by an accelerometer inside a smartphone positioned on a specific part of the body. The worst mean coefficient of symmetry was found for exercise E6, with a mean value of 3.4 and the highest standard deviation of 2.4, indicating the difficulty of this rehabilitation motion for all participants. Exercises E1, E2, E5, and E7 were among the easier ones, with their coefficients of symmetry below 3 and standard deviations below 2.

The classification accuracy reached 90.6% for the two-layer neural network, with a cross-validation error of 0.11 (using the leave-one-out method) for individual 6-DH, who had the highest asymmetry and a coefficient of symmetry of 7, as shown in Figure 7. Rehabilitation specialists must also consider individuals’ ages, as some exercises are complicated for elderly people, and their physical condition can affect the performance of rehabilitation exercises.

Integrating sensor technology into pre-operative and post-operative care could help develop a more sophisticated data-driven approach to surgical planning, monitoring, and follow-up, improving outcomes and advancing the standards of surgical care. Sensors can continuously track key indicators, such as range of motion and muscle strength. Early signs of complications can be detected through subtle changes in these indicators.

Limiting factors of the use of accelerometers for the evaluation of rehabilitation symmetry include sensitivity to the placement of sensors, sensitivity to external factors (like uneven surfaces), congenital asymmetry of movement, age of patients, and data interpretation. The influence of the last item can be reduced by correctly selected signal processing methodology and AI application.

Evaluation of the rehabilitation exercises can be conducted through the proposed web-page to inform individuals about their progress and to motivate them to perform rehabilitation exercises more precisely. Future studies should focus on more complex computational methods and the use of multichannel sensor systems for time-synchronized monitoring of motion patterns, allowing for a more detailed analysis of rehabilitation exercises. These applications will include the use of more sophisticated sensors, advanced computational methods, and deep learning strategies to monitor rehabilitation patterns, potentially utilizing augmented reality and telerehabilitation.

## 5. Conclusions

The integration of mobile accelerometers and computational analysis in rehabilitation exercises has the potential to significantly enhance healthcare and fitness levels. The comprehensive data from appropriate sensors can lead to more personalized and data-driven decision-making, improving patient outcomes. To realize the full potential of mobile accelerometers in rehabilitation care, further research and collaboration between healthcare professionals and technology developers are critical.

There is a high risk of developing post-operative complications after abdominal surgery for patients with lower pre-operative physical activity. Hence, pre-operative specific rehabilitation and physical activity measurement may be useful in decreasing post-surgery complications.

The future of computational intelligence in the analysis of rehabilitation exercises is promising, with the potential to significantly enhance the precision, personalization, and effectiveness of rehabilitation programs. An emerging trend involves using wearable sensors and digital technology to monitor motion activities. By integrating advanced technologies such as machine learning, computer vision, and robotics, rehabilitation can become more adaptive, patient-centered, and efficient, ultimately leading to better outcomes and an improved quality of life for patients undergoing surgery.

In rehabilitation settings, the use of accelerometers is becoming more prevalent due to their affordability, ease of use, and integration with other technologies. This makes them a key tool in improving the quality and effectiveness of rehabilitation therapy.

## Figures and Tables

**Table 1:** Description of selected exercises used for prehabilitation before the surgery treatment.

Exercise	Name	Description
E1	basic spinal motion	both legs bent
E2	spinal motion	one leg bent
E3	lifting of one leg	other leg on the floor
E4	foot circles	circles in the hip joint
E5	arm flection	arms motion
E6	body cross-motion	body sculpture rotation
E7	leg lifting	one-leg lift
E8	squat	high squat

**Table 2:** Description of participants in the rehabilitation exercises, including age, gender, height, and BMI of each individual.

Individual	Age [year]	Gender m/f	Height [cm]	BMI [kg/m^2^]
1-AP	75	m	187	27.7
2-HCH	45	f	152	21.6
3-AM	21	f	173	18.0
4-DM	21	m	184	21.6
5-DDM	47	m	178	26.5
6-DH	24	m	185	22.8
7-JH	21	m	176	22.3
8-JM	69	m	185	27.5
9-LN	22	m	182	19.0
10-VM	47	f	163	25.6
11-MS	34	m	192	27.1
12-AB	47	m	176	22.6
13-TT	22	f	175	24.5
14-KA	8	f	135	21.6
15-T2	22	f	175	24.5
16-H2	46	f	152	21.6
MEAN	35.7		173.1	23.4
STD	18.9		15.3	2.9

**Table 3:** Results of symmetry values of the set of 16 tests of different individuals (Ind) for separate exercises evaluated by the mixed-domain features, and their mean values associated with each participant of the study.

*Ind.*	Exercise								Mean
	E1	E2	E3	E4	E5	E6	E7	E8	
1	1.5	1.7	2.1	2.4	2.3	7.7	1.0	5.7	3.0
2	1.5	3.7	4.5	2.1	1.1	0.6	2.9	3.7	2.5
3	2.0	0.8	1.9	3.2	3.5	7.3	1.6	1.6	2.7
4	3.0	0.8	1.8	1.4	0.3	3.5	3.5	3.3	2.2
5	3.1	6.2	6.7	3.8	3.8	2.9	1.6	0.6	3.6
6	4.8	3.6	5.1	5.8	3.6	7.0	1.3	3.0	4.3
7	0.5	0.7	1.5	3.9	2.5	1.3	0.8	1.9	1.6
8	3.9	3.1	3.7	0.8	3.2	3.8	4.2	7.6	3.8
9	2.9	4.8	5.9	4.7	5.9	2.5	1.5	0.9	3.6
10	2.5	5.7	0.8	5.6	0.1	4.0	2.3	0.5	2.7
11	0.1	3.9	4.7	2.3	2.9	1.2	1.6	4.4	2.6
12	1.3	2.5	1.8	1.1	2.0	0.8	4.5	3.0	2.1
13	0.7	3.5	0.8	4.0	1.3	4.7	1.8	1.6	2.3
14	4.3	0.6	1.4	7.5	3.6	4.6	7.3	3.4	4.1
15	1.0	1.0	2.0	1.0	1.3	0.4	0.8	1.3	1.1
16	1.4	1.7	4.0	3.6	0.6	1.7	1.3	5.5	2.5
Mean	2.1	2.8	3.0	3.3	2.4	3.4	2.3	3.0	
Std	1.4	1.8	1.9	1.9	1.6	2.4	1.8	2.0	

**Table 4:** Symmetry classification of rehabilitation exercise patterns performed by the support vector machine, Bayesian, and two-layer neural network methods using two features specified as the power in the selected frequency band and the associated standard deviation of accelerometric data, presenting the accuracy (AC) and the cross-validation error (CV) for exercise 6 and all individuals (Ind).

*Ind.*	SVM Method		Bayes Method		NN Method	
	AC [%]	CV	AC [%]	CV	AC [%]	CV
1	69.6	0.39	59.8	0.37	76.1	0.34
2	72.0	0.42	54.8	0.53	76.3	0.16
3	84.9	0.25	79.6	0.28	84.9	0.16
4	63.4	0.45	54.8	0.56	66.7	0.44
5	76.8	0.37	73.7	0.23	82.1	0.18
6	86.5	0.21	81.3	0.25	90.6	0.11
7	72.6	0.38	71.6	0.35	72.6	0.21
8	63.4	0.45	59.1	0.48	74.2	0.25
9	66.3	0.40	64.1	0.47	78.3	0.30
10	73.7	0.39	63.2	0.40	75.8	0.20
11	68.1	0.44	52.7	0.48	69.2	0.22
12	60.9	0.48	52.2	0.47	72.8	0.23
13	68.5	0.46	64.1	0.34	70.7	0.35
14	71.4	0.31	72.5	0.33	81.3	0.21
15	69.1	0.43	50.0	0.39	66.0	0.39
16	72.3	0.33	67.0	0.38	76.6	0.32

## Data Availability

Accelerometric data acquired during all experiments with detailed descriptions of observations can be found on IEEE DataPort (Rehabilitation Exercises and Computational Intelligence, 10.21227/xp41-7325) for further investigation (https://doi.org/10.21227/xp41-7325, accessed on 11 November 2024).
